# A Bureau of Information

**Published:** 1914-03-21

**Authors:** 


### A BUREAU OF INFORMATION.

The object of this Bureau will be to render practical aid and advice to doctors' wives on many matters which may have a special interest for each. The following branches which it will aim to cover will sufficiently indicate what it is hoped to provide.

The Science of Holiday-making.—We have already an organisation with an adviser on travel and holiday questions. It is possible, with understanding, if directed by knowledge and experience, as Madame Vallois properly insists, to make foreign travel cheaper than home travel, and it is fuller of interest and change. Most English people who take a holiday put themselves to greater expense and reap less benefit from it than they might if they would think of their plans in good time, consult the Bureau, and so enable it to supply the necessary information and to secure the best results. Everyone should begin to make inquiries not later than the first week in May, and the earlier the better. This is a point which should not be forgotten, especially where the aid of the Travel Agency of the Bureau is desired.

Careers for Sons and Daughters.—Many mothers and fathers often have great difficulties in deciding upon a career for a son or daughter. When a decision is come to the difficulties increase, because an opening has to be found. Here it is hoped the Bureau may sometimes be able to render encouraging help.

Hobbies as Yielding Income.—The staff of the Bureau will have upon it experts in the rearing of fowls, the keeping of bees, the breeding of dogs, and other methods whereby a hobby can often, with intelligent industry and hard work, be made to yield a helpful income.

Women's Wants and Views.—The Editor of the *Doctor's Wife* will welcome resort to its columns by all members of the Association who have special wants and can tersely and intelligently explain them. A column or more will be devoted to correspondence, in which the doctor's wife will have an opportunity to express her views upon matters which are calculated to prove of interest not only to herself, but to members of the Association generally.

